# Study on Preparation of Low Heat High Belite Clinker from Waste Mortar and Its Modification

**DOI:** 10.3390/ma15093196

**Published:** 2022-04-28

**Authors:** Zhouxiang Yang, Xu Tao, Guoling Wang, Weifeng Li

**Affiliations:** College of Materials Science and Engineering, Nanjing Tech University, Nanjing 211816, China; 201961203129@njtech.edu.cn (Z.Y.); 202061203251@njtech.edu.cn (X.T.); 201961103078@njtech.edu.cn (G.W.)

**Keywords:** waste mortar, high belite clinker, SO_3_, mineral composition, physical properties

## Abstract

In order to realize low energy consumption in cement and the recycling of solid waste, the phase composition and structure of waste mortar used to prepare a high belite cement clinker, instead of some other raw materials, have here been investigated, and the belite was activated by doping with SO_3_. The results show that a good high belite cement clinker can be obtained using waste mortar, limestone, coal gangue, iron powder, or iron correction raw materials combined at 1350 °C for half an hour. The addition of SO_3_ greatly increased the strength of the clinker in the early phase, and overall, the ratio of calcium to silicon in belite became higher, and more Al_2_O_3_ entered the belite; however, the generation of C_3_S was inhibited somewhat, and the content of C_4_AF also increased. This study offers guidance for the application of waste mortar and the activation of belite, which offers huge environmental and economic benefits.

## 1. Introduction

Since the beginning of the century, continuous processes of demolition and construction have produced an incalculable amount of construction waste. As the largest component of construction waste, waste concrete is usually piled up and buried, which not only exasperates the shortage of landfill land but also wastes resources; this necessitates the application of waste concrete.

In general, the simplest resource-oriented mode of utilization is breaking the material after using it as filling [1] or isolating the broken recycled aggregate instead of the natural aggregate concrete [2]. When used as a filling material, waste concrete contains many impurities, which makes it difficult to treat and use. The surface of the separated recycled aggregate contains some cement mortar, resulting in a high water absorption rate and poor physical properties, such that the prepared concrete has low strength and poor durability. In recent years, fine powders, primarily made of hardened cement stone, have been used in screening waste mortar. The C-S-H gel and CH present in hardened cement stone are dehydrated by calcination at high temperatures in order to recover the hydration activity, and these are then used as regenerative cementing materials. However, this method has some limitations: the amount of primary cement that is separated is relatively small; it is difficult to separate hardened cement slurry from waste concrete; a large quantity of free calcium oxide is formed after CH dehydration, which leads to excessive mixing water; and the strength of the prepared cementing material is low [3,4,5,6].

One favorable method involves using waste concrete in cement raw material preparation [7,8,9]. In recent years, many scholars have studied the recycling of waste mortar. Waste limestone coarse aggregate can provide calcium oxide as the required calcium material, and siliceous fine aggregate can be used as the siliceous material to provide silica, resulting in a hardened mortar that retains its hydration activity under high temperatures [10]. Here, there is no need to screen the waste concrete, the utilization cost is low, and the utilization rate is high. In this way, we can realize the closed-circuit circulation of cement–concrete–cement as well as the high value-added utilization of construction waste, and alleviate the resource crisis within the cement industry. This is thus an effective way to ensure the sustainable development of the cement concrete industry.

Portland cement clinker is known to require a large amount of energy and produces large amounts of CO_2_ [11]. The strategies developed to address these issues include the use of alternative fuels and the supplementing of cementing materials [12,13,14]. The synthesis of belite cement as a way to save energy and raw materials has attracted much attention in recent years. Belite Portland cement typically contains more than 50% C_2_S, and requires less CaO than OPC (which is mainly C_3_S), thus reducing the carbon emissions of the production process [15]. The clinker temperature of belite cement is about 100 °C lower than that of OPC, which reduces the CO_2_ generated by the burning of fuel and reduces the energy consumption of the clinker [16]. In theory, the energy required to produce belite is about 1350 kJ/kg, while alite requires about 1810 kJ/kg [17].

In addition, industrial activities produce wastes that are best treated according to their chemical composition, which can lead to the production of many substitutes for the raw materials (clay and limestone) currently used in cement production. Martinez et al. [18] prepared belite clinkers containing more than 60% calcium silicate from ceramic and marble waste; Maria et al. [19] prepared a belite clinker at 1250 °C from wastes of the pulp and paper industry, as well as steel slag and quart stone mining tailings, which performed well. The recycled belite cement prepared by this method was mixed with Portland cement at 10% by weight and showed good performance. Xiansu et al. [20] prepared a high-strength belite Portland cement clinker with about 30% alite content by mixing limestone, quartz sand, low-alkali shale, bauxite waste rock, copper slag, and gypsum; after the addition of an appropriate amount of gypsum, the high-strength and high belite Portland cement obtained after 7 d had a compressive strength greater than 22 MPa, and after 28 d, greater than 54 MPa. However, a major disadvantage of Beret cement is its low reactivity and poor strength in the early hydration phase due to its high thermodynamic stability and dense structure, which prevents water from eroding it [21]. Over the years, researchers have used various methods to increase the activity of cement. Karakouzian et al. [22] added different weights of nano-silica and carbon nanotubes to cement paste to improve its mechanical properties. The addition of nano-silica improved the compressive strength of cement paste. The improvement rates of cement slurry at 7 and 28 days of age were 111% and 117%. Although the strength of cement slurry can be reduced by adding nano-silica and carbon nanotubes simultaneously, the flexural strength can be greatly increased—by 182% at 7 days. Borrero et al. [23] studied the durability and mechanical properties of concrete under different curing systems and identified the maximum compressive strength. In response to the problems posed by high belite cement, increasing numbers of experts and scholars are researching mineral belite, and have improved the early-phase strength of belite by improving its hydration activity. Morsli et al. [24] studied the effects of compound doping different amounts of Na_2_O, K_2_O, and SO_3_ on belite, and found that the addition of alkaline cations and sulfur can stabilize α’-C_2_S, thus producing a belite clinker. Yanting Z et al. [25] found that the presence of SO_3_ can effectively stabilize β-C_2_S, while the addition of BaO and MgO can stabilize α’-C_2_S, a-C_2_S, and β-C_2_S. All this proves that it is feasible to prepare a belite clinker from waste materials.

In this research, waste mortar sieved from waste concrete abandoned over 2 years ago was used as the raw material to replace some of the siliceous and calcium raw materials used to prepare belite clinker, and we expected to increase the early-phase strength by adding SO_3_ [26]. Via our systematic research on the modification of belite with SO_3_, we hope to offer guidance in the utilization of waste concrete, and also provide technical support for the application of belite clinkers.

## 2. Experimental Section

### 2.1. Raw Materials

**Figure 1:**
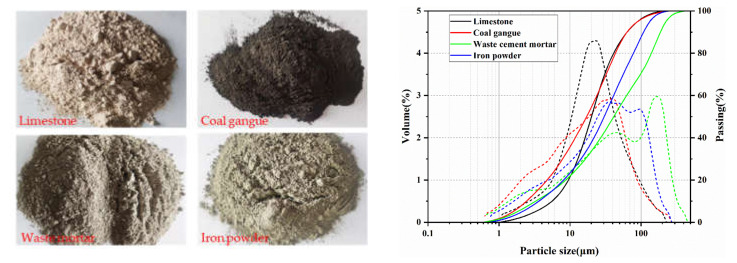
Raw materials and the particle size distribution of raw materials. (Solid line: pass rate Dashed line: volume ratio).

The raw materials used in our preparation were pure limestone with 51.65 wt.% CaO, coal gangue with 51.02 wt.% SiO_2_ and 21.65 wt.% Al_2_O_3_, and waste cement mortar with 70.52 wt.% SiO_2_ and 9.65 wt.% CaO (derived from waste concrete separated from aggregate); low-grade iron powder and analytically pure iron chemical reagents served as the calibration materials, and the iron chemical reagents and analytically pure chemical reagents CaSO_4_∙2H_2_O and CaSO_4_∙2H_2_O were produced by Nanjing Wanqing Company (Nanjing, China). The specific chemical components are shown in Table 1. Figure 1 shows the raw materials and their particle size distributions. The average particle sizes of limestone, waste cement mortar, coal gangue, and low-grade iron powder were 20.1 µm, 41.43 µm, 17.21 µm, and 30.38 µm.

In this study, three parameters were used to control the expected composition of the cement clinker, with the following equation [27]:

(1) $$\begin{array}{c} \mathrm{SM}=\frac{{\mathrm{SiO}}_{2}}{{\mathrm{Al}}_{2}O_{3}{+\mathrm{Fe}}_{2}O_{3}} \end{array}$$

(2) $$\begin{array}{c} \mathrm{IM}=\frac{{\mathrm{Al}}_{2}O_{3}}{{\mathrm{Fe}}_{2}O_{3}} \end{array}$$

(3) $$\begin{array}{c} \mathrm{KH}=\frac{\mathrm{CaO}-{1.65\mathrm{Al}}_{2}O_{3}-{0.35\mathrm{Fe}}_{2}O_{3}}{{2.8\mathrm{SiO}}_{2}} \end{array}$$

where KH is the limestone saturation coefficient, SM is the silica modulus and IM is the aluminum modulus.

The composition and chemical parameters of the raw materials are given in Table 2, Table 3 and Table 4. This experiment intended to prepare a belite clinker with different contents of C_2_S and C_3_S, control the increasing KH in the three clinkers and keep the SM, IM, and mesophase content of the clinker as unchanged as possible, in order to address the influences of the different contents of C_2_S and C_3_S on the performance of the clinker. The KH values of the clinkers in groups A, B, and C were the same. Group B used an iron correction raw material to ensure that the amount of waste mortar was greater than that in Group A. Group C was supplemented with more SO_3_ than group B, so as to determine the content of waste mortar, and the influence of SO_3_ on the belite clinker.

**Figure 2:**
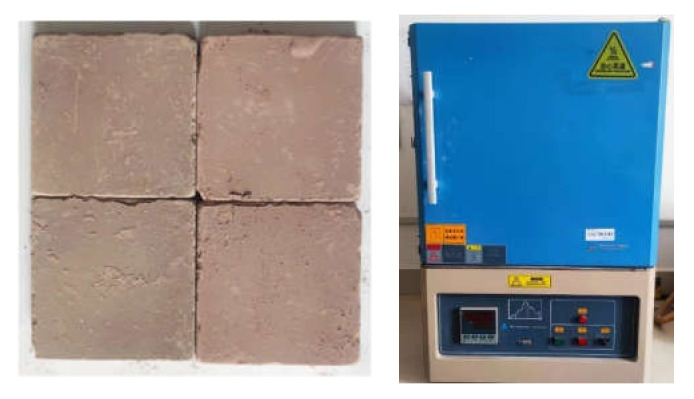
Block cement raw material and high-temperature furnace.

The raw materials were pressed into tablets with side lengths of 4 cm, weighing about 50 g. All belite clinkers were burned in a high-temperature furnace (kejing, Hefei, China) in the following regime: rate of temperature rise 10 °C/min, calcination at 1350 °C for half an hour. After calcination, the samples were removed and cooled to room temperature by wind blowing. The block cement raw material and electric furnace are shown in Figure 2.

### 2.2. Measurement Methods

The METTLER TOLEDO 1600LF (METTLER TOLEDO, Zurich, Switzerland) comprehensive thermal analyzer was used in this test. N_2_ was used as a protective gas and the heating rate was 20 °C/min. The sample was placed in an α-Al_2_O_3_ crucible and the test temperature range was 25–1400 °C.

A Rigaku SmartLab 3000A X-ray diffraction instrument (Rigaku, Tokyo, Japan) equipped with a copper target (CuKα, λ = 0.154 nm) was used to measure and analyze the mineral compositions of samples. The working voltage and current of the XRD tests were 40 kV and 20 mA; the scanning speed and scanning range were 5–70° and 5°/min, and the step size was 0.01.

Search–Match software (OXford Cryosystems Ltd., Oxford, England) was used to identify all mineral phases in the test samples, and the PDF card number corresponding to each mineral was recorded. Find IT software2011 (The Gmelin Institute, Frankurt, Germany) was used to find the CIF cards of all mineral phases using the PDF card number. The relevant information of the CIF cards of the mineral phases used in this system is shown in Table 5.

Then, High Score Plus software3.05 (Malvern Panalytical, Marvin, England) was used to refine the collected XRD patterns. The refining parameters were the scale factor, unit cell, and profile variables. Rwp and Gof are statistical parameters indicating the quality of the Rietveld fitting, which values are less than 10 and 2, respectively, in the fitting process. The scale factor, density, and volume of each phase were obtained via map fitting. µα could be calculated by inputting the content of each oxide in the sample through the tool function of the software [28].

A Nicolet-IS5 spectrometer (Lijing Scientific Instruments Co., Shanghai, China) was used to conduct Fourier transform infrared (FT-IR) spectroscopy from 400 to 4000 cm^−1^, with KBr as the standard material.

An 8-channel isothermal calorimeter (TAM Air; Thermometric AB, Jarfalla, Sweden) was used to determine the heat evolution during sample hydration. The measurement method of internal agitation was adopted. We accurately weighed 2 g samples in glass ampoules. Then, we used a needle tube to accurately weigh 2 g of distilled water and insert this into the microcalorimeter, together with the sample bottle. After the machine reached the baseline balance, the syringe was pressed down, quickly filling the ampoule with water, and we then manually rotated the mixture for 2 min. The heat flow curves of the samples have been recorded for 72 h at a constant temperature of 20 °C.

The phase assemblage of the clinker was studied on a Zeiss Ultra55 field (Rigaku, Tokyo, Japan) emission scanning electron microscope with a W-filament. The backscattered scanning electron (BSE) mode was chosen, and the accelerating voltage was 15 kV. The chemical composition of the microscopic clinker phases was determined by energy-dispersive X-ray spectroscopy (EDS) using an X-max 50 X-ray energy spectrometer. The EDS results of wt.% oxide were recast into a composition formula calculated in atomic units, according to the method described in [34].

**Figure 3:**
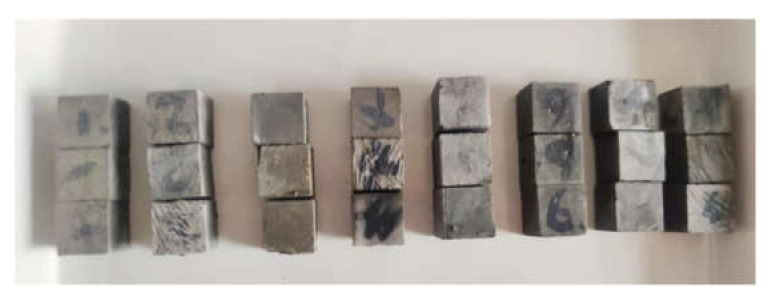
Cement slurry test block.

Finally, 6 wt.% of gypsum was added to all the belite clinkers to produce cement samples with similar specific surface areas. The compressive strength of the paste (Square, 2 × 2 × 2 cm, as shown in Figure 3) was measured with servo-hydraulic compressor after curing for 3, 7, 28, 56, and 90 days, and the compression test was carried out according to GB/T17671-99 [35].

## 3. Results and Discussion

### 3.1. Composition of Clinkers

**Figure 4:**
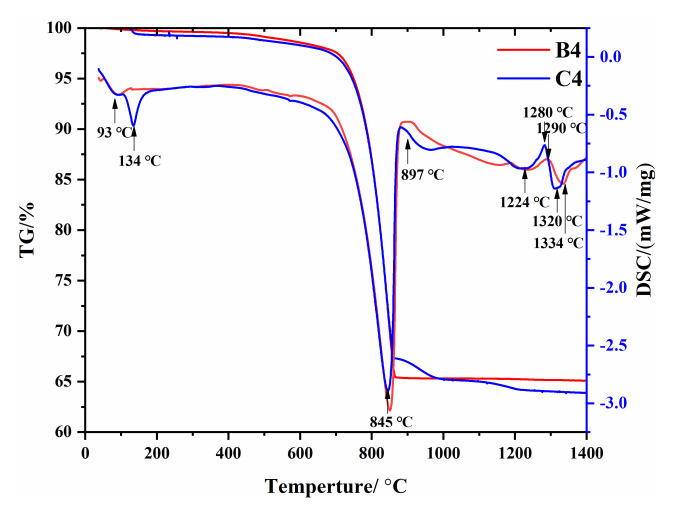
TG-DSC curves of B4 and C4.

The TG curves of samples B4 and C4 are shown in Figure 4. In general, the weight loss induced at temperatures between 30 and 200 °C resulted from the elimination of weakly bound water (dehydration). The slow reduction in weight between 200 and 700 °C resulted from the elimination of organic matter and the dehydroxylation of silicates. The weight loss between 700 and 870 °C was due to the decomposition of CaCO_3_. According to the DSC analysis, once the CaCO_3_ was completely decomposed following the heating process, the CaO began to react with the silicate and aluminosilicate in the temperature range of 897–1200 °C, and the reaction zone was the endothermic zone. There were no significant peaks in this range because these solid-state reactions were diffusion-controlled and exhibited flat and wide frequency bands. As the temperature continued to rise, exothermic spikes were seen at 1280 °C and 1290 °C due to the solidification of the liquid phase.

Endothermic peaks emerged at 1320 °C and 1334 °C due to the formation of the molten phase (clinkering). This is consistent with the results of Trezza [36]. In addition, according to Staněk’s study [37], the DSC curve of C4 with SO_3_ emerged at 850~1200 °C, probably due to the decomposition of gypsum.

**Figure 5:**
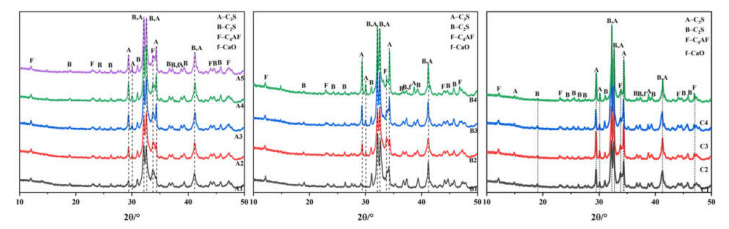
XRD patterns of samples sintered 1350 °C, for 30 min.

**Figure 6:**
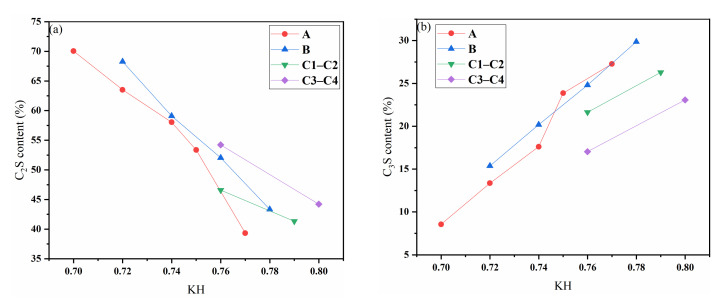
Diagram of phase variation with limestone saturation coefficient (**a**) C_2_S (**b**) C_3_S.

The XRD patterns of samples sintered at 1350 °C for 30 min are shown in Figure 5, and the dotted line shows the diffraction peaks undergoing obvious change. The phase compositions are listed in Table 6, Table 7 and Table 8. The contents of C_3_S, C_2_S, and C_4_AF in the clinker are consistent with the mineral composition inferred from the XRF data. No diffraction peak of free-CaO could be clearly seen, while the diffraction peaks of C_3_S and C_2_S were present at 1350 °C, indicating calcination of the clinker at 1350 °C. The content of C_3_S increased with the increase in KH, and the C_2_S decreased accordingly. Because the SM was larger (about 2.7) and the IM was smaller (about 0.86), the amount of C_3_A generated was lower, and the diffraction peaks were not obvious; this is because a certain amount of Al_2_O_3_ will be solubilized in C_2_S and the content of C_3_A will be reduced, while some C_3_A may be solubilized in the glass phase [37]. As Figure 6 shows, with basically the same rate values, the amount of C_3_S generated in group B (with more waste mortar) is much higher than in group A, because there is a certain content of hardened cement slurry in the waste mortar, and minerals with hydration properties, such as C_2_S, C_12_A_7,_ and C_4_AF, can form after calcining [38]. These act as crystal seeds, while the waste concrete in group B is greater than that in group A. Therefore, in the same period, the amount of C_3_S in group B was higher than that in group A. This shows that the addition of waste mortar is beneficial to the calcination of cement clinker. The content of C_3_S was lower, and that of C_2_S higher, in the samples supplemented with SO_3_. The addition of SO_3_ obstructs the generation of C_3_S, and stabilizes C_2_S. Meanwhile, the content of C_4_AF in the samples supplemented with SO_3_ was also higher, which is consistent with Li’s [39] research.

**Figure 7:**
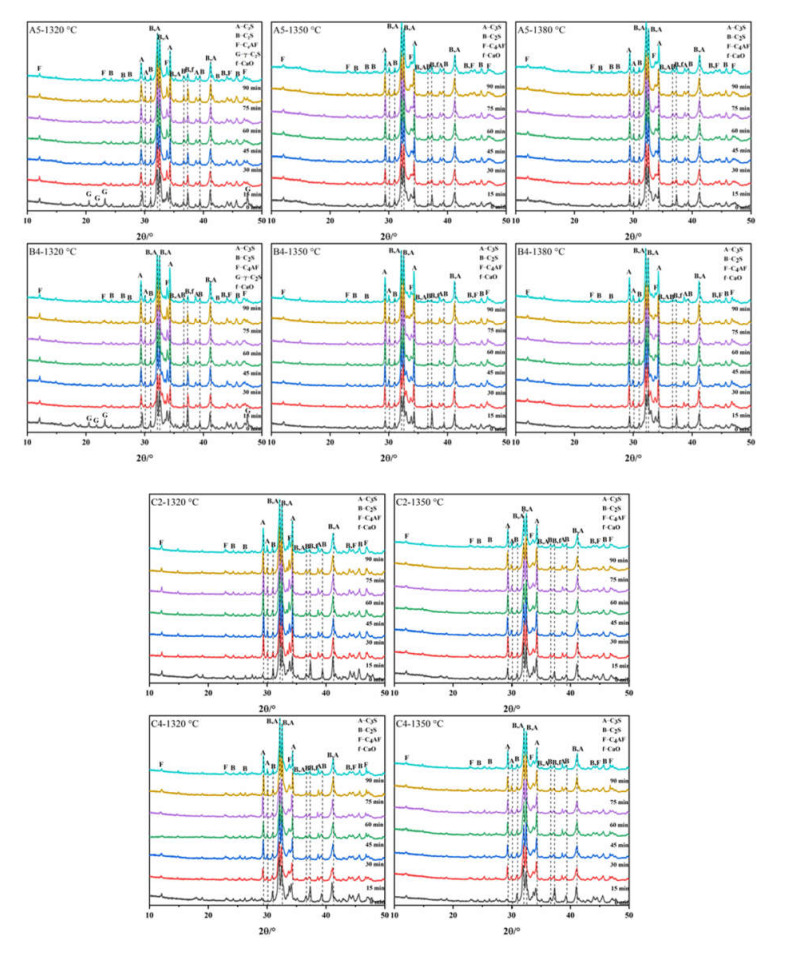
XRD of different clinkers at different calcination times and temperatures.

In order to study the influence of calcination temperature and holding time on the synthesis of clinker using waste concrete as the raw material, four groups of raw materials with similar rates of A5, B4, C2, and C4 were specifically selected. As all the samples contained the same raw materials but in different proportions, the KH, SM, and IM of the A5, B4, C2, and C4 samples were sufficiently close to show the regularity of this clinker. These samples were heated to 1320 °C, 1350 °C, and 1380 °C, respectively, and held for 0, 15, 30, 45, 60, 75, and 90 min. The sintered samples were analyzed by XRD and Rietveld refinement. Figure 7 shows the XRD results of calcination. At 1320 °C, a large number of γ-C_2_S and CaO diffraction peaks emerged. The reaction between CaO and C_2_S in the clinker was not complete, and the content of belite in the clinker was too high, meaning the belite could not undergo rapid crystal transformation during rapid cooling from β-C_2_S (with hydration properties) to γ-C_2_S (without hydration properties). Under the conditions, volume expansion will occur and lead to clinker pulverization [40]. However, at 1350 °C and 1380 °C, no obvious γ-C_2_S diffraction peaks emerged. This was because the increase in temperature led to an increase in C_3_S production and a decrease in belite activity [41]. The XRD results show that the sintering range of the high belite cement prepared from waste mortar was wide.

**Figure 8:**
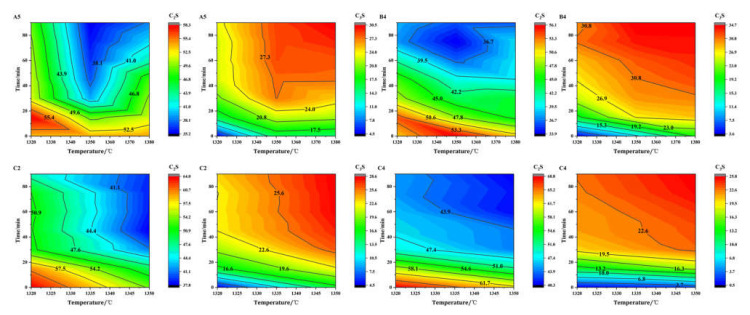
Contours of C_2_S and C_3_S contents in different clinkers with calcination time and temperature.

The sintered samples were analyzed by XRD and Rietveld refinement. Figure 8 shows the contours of C_2_S and C_3_S contents in different clinkers, which varied with calcination time and temperature. Since γ-C_2_S, with no hydration activity, was present in groups A5 and B4 at low temperatures, the contour diagram of β-C_2_S with hydration properties exhibits a ring profile at 1320 °C. Furthermore, the content of β-C_2_S decreased first and then increased with the increase in holding time. With the increase in calcination temperature and sintering time, the content of C_3_S increased gradually, and the content of C_2_S in each sample showed a trend of first increasing and then decreasing. The high belite cement showed a wide sintering range and could calcinate clinker with less free CaO at 1320~1380 °C. The addition of waste concrete does not lead to the generation of other mineral phases, but increases the content of C_3_S because it acts as a seed crystal.

The composition of the high belite cement clinker mixed with SO_3_ was the same as that of group B without SO_3_, showing the same trend, and there was no characteristic peak of calcium sulfoaluminate, which may be due to the lower IM and alumina contents in the set ratio. There was no γ-C_2_S when the calcination temperature and holding time were lower because the β-C_2_S was stabilized by the addition of SO_3_. At the same calcination temperature and sintering time, the rate of C_3_S production in group C was significantly lower than that in groups A and B, while more C_2_S was produced in this group than in groups A and B. This is because the increase in SO_3_ prevents the generation of C_3_S and stabilizes C_2_S. Andrade’s study [42] also showed the shrinkage of the C_3_S stability field, and the preferential uptake of sulfur by β-C_2_S, which thus stabilizes β-C_2_S and prevents the formation of γ-C_2_S. The higher the SO_3_ content, the higher the calcium–silicon ratio, and the higher the required Ca^2+^ content, the lower the C_2_S content. In general, the content of C_2_S decreases with increases in calcination temperature and holding time, and the content of C_3_S increases with increases in calcination temperature and holding time. The sintering range of belite cement is relatively wide. When used as a raw material, waste mortar acts as part of the seed crystal, which aids in the sintering of cement clinker, while the addition of SO_3_ slows down the generation rate of C_3_S and increases the content of C_2_S.

**Figure 9:**
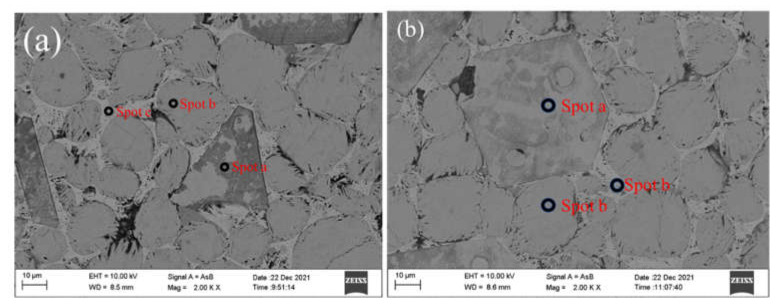
Microstructure of belite-rich clinker (**a**) B4 (**b**) C4.

Figure 9 shows BSE images of the high belite cement clinker. The alite shown in Figure 9a has euhedral crystal outlines, while the crystal size of alite is small, which indicates that alite is not over-burned in the formation of large crystals, resulting in reductions in the hydration activity and strength of the cement clinker [43]. The belite shown in Figure 9b is round and elliptical, with a smooth surface and a high content. In the intermediate phase of c, there is an even distribution between alite and belite, showing the lightest and smoothest color. In Figure 9b, we see belite inlaid in the middle of the alite, indicating that the synthesis of alite is dominated by belite here, whereby CaO gathers around the belite and gradually forms alite.

The chemistry of the belite grains was determined by electron microanalysis, which also shows the total charge of the cations and skeleton elements. The results are given in Table 9, where it is assumed that all Mg is located at the Ca site and all Fe is trivalent, containing the Si site. The calculations of the atomic ratios of elements filling the Ca site. (i.e., Ca, Mg, Na, K) versus those filling the Si site (Si, S, P, Al, Fe^3+^, Ti) show that they are close to stoichiometric 2:1, while the Ca/Si ratio of the belite supplemented with SO_3_ is 2.3~2.6. The belite Ca/Si ratio found during calcination experiments performed on sulfur-rich raw materials by Herfort et al. ranged from 2.37 to 2.42 [44]. The average atomic ratio values of the belite components shown in Table 10 indicate a statistical correlation between the various elements (α = 0.05). The significant negative correlations of Si with Al and S (Pearson’s r = −0.897 and −0.929), and the significant positive correlation between Al and S (Pearson’s r = 0.77), indicate that the higher the content of Al and S in belite, the lower the content of Si, and the Al and S increase synchronously, indicating the occurrence of Si ↔ Al + S substitution [45,46]. Staněk et al. [37] reached the same conclusion.

**Figure 10:**
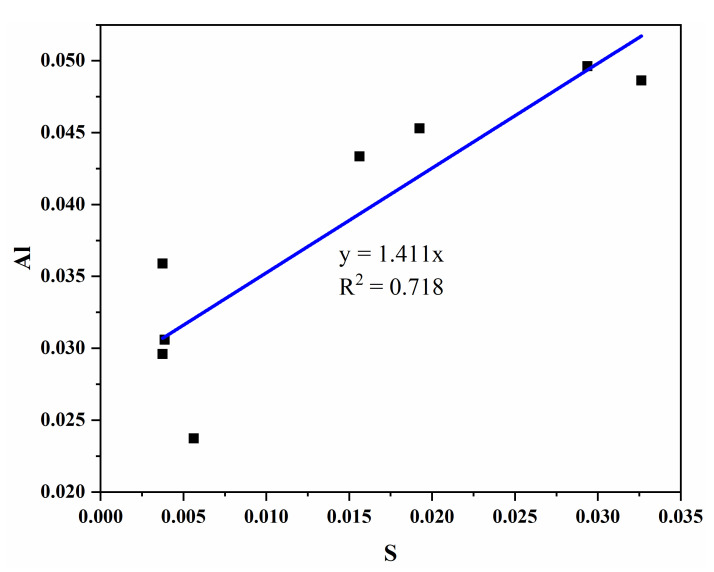
Relationship between the average content of sulfur and aluminum in belite.

Figure 10 illustrates the microscopic regression analysis of belite when replacing SiO_4_ groups with AlO_4_ and SO_4_; here, the atomic ratio of Al/S is close to 1.5:1 (average 1.411). In the study of Bonafous [47], 3[AlO_4_]^5−^ and 2[SO_4_]^2−^ replaced 5[SiO_4_]^4−^. The equation is

5[SiO_4_]^4−^ = 3[AlO_4_]^5^^−^ + 2[SO_4_]^2^^−^ (4)

The solution of S^6+^ will promote the solution of Al^3+^, and the solution of S^6+^ and Al^3+^ will change the crystal structure of C_2_S when in 4-fold coordination (r(Si^4+^) = 0.26 nm, r(Al^3+^) = 0.39 nm). When Al^3+^ replaces Si^4+^ and enters the silicon-oxygen tetrahedron, the ionic radius of Al^3+^ will be larger than that of Si^4+^, which will expand the space occupied by the silicon–oxygen tetrahedron. The adjacent Ca–O octahedron is indirectly deformed, and the space becomes smaller. Our study has shown that the sulfur-rich belite clinker had higher Ca/Si, leading to more Al entering belite, which leads to a reduction in C_3_A and C_3_S.

### 3.2. Cement Properties

**Figure 11:**
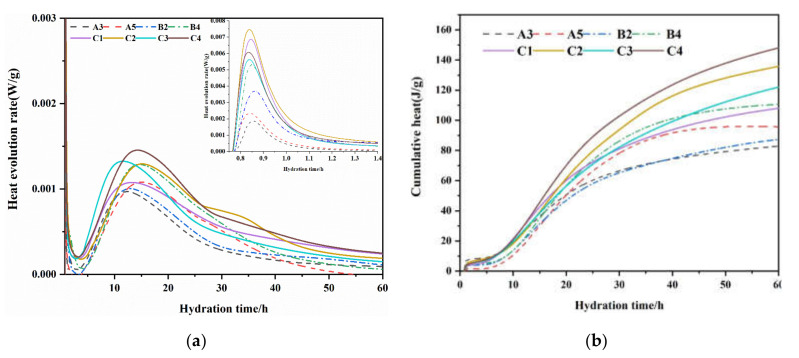
Heat release of composite system (**a**) heat evolution rate (**b**) cumulative heat release.

Figure 11 shows the hydration heat release of the belite clinker prepared from waste cement mortar. The cumulative heat release of all samples was less than 180 J/g, which satisfies the requirements of low-heat cement.

In Figure 8, the high belite cement shows two exothermic peaks; the first, between 0.8 h and 1.4 h, mainly concerns the rapid hydration of C_3_A, the initial precipitation of hydrates, and the wetting of the system; the second, between 10 and roughly 20 h, mainly concerns the hydration of C_3_S, C_4_AF, and active C_2_S. The sample with high alite content showed a higher hydration heat release rate and cumulative heat release. Among the different samples, the hydration heat release rate and cumulative heat release rate of group B were higher than those of group A, with the same rate value. This is because there was more waste mortar in group B, resulting in more alite. This shows that the addition of waste mortar is beneficial to the strength of high belite cement. Group C, doped with SO_3_, showed the largest hydration heat release rate and cumulative heat release. Although the addition of SO_3_ had an inhibitory effect on the generation of C_3_S, according to Xuerun L’s [39] research, SO_3_ can not only activate belite, but it also stabilizes the M1-type alite with a higher hydration activity, meaning samples doped with SO_3_ themselves show a higher hydration activity.

**Figure 12:**
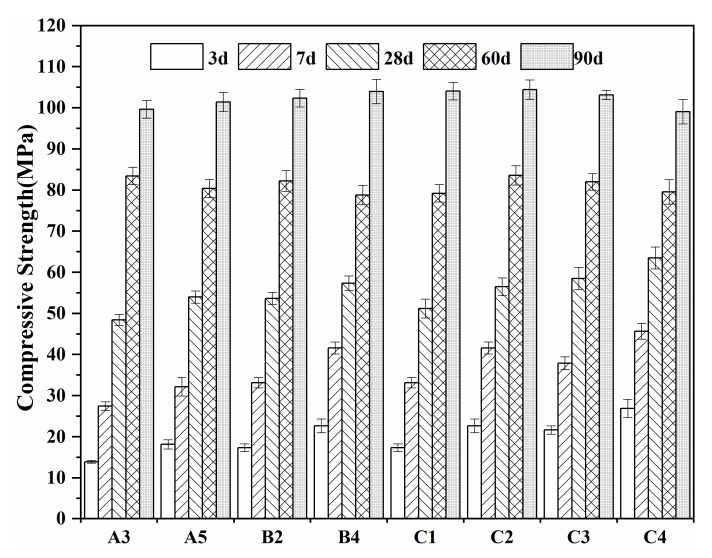
Compressive strength of A3, A5, B2, B4, C1–C4.

Figure 12 shows the strengths of the A3, A5, B2, B4, and C1–C4 cement clinkers at 3, 7, 28, 56, and 90 d. The strength of the sample prepared from waste mortar without SO_3_ was 13–18 MPa at 3 d, and 48–57 MPa at 28 d. At the same hydration age, the early-phase strength of group B was higher than that of group A, because the mortar content of group B was higher, such that the seed crystal effect was more obvious, and more alite was produced. This indicates that the addition of waste mortar was conducive to the increase in the early-phase strength of belite cement. The 3 d strength of the sample prepared from SO_3_-mixed waste mortar was 17–26 Mpa, and at 28 d it reached 51–63 MPa. The early-phase strength of the sulfur-rich belite clinker was significantly higher than that of the SO_3_-free clinker, indicating that the higher the calcium–silicon ratio, the faster the hydration of belite. At the same time, using more Al solution will also increase the activity of the belite. The addition of SO_3_ improves the early-phase strength of the belite clinker. Compared to pure C_2_S, the clinker prepared from a waste mortar instead of silicon source showed greater early-phase strength growth when it contained 20% to 30% alite. SO_3_ can stabilize monoclinic M1-type alite, and alite formation also improves the parameters of burning and grindability. After 56 days, the SO_3_-doped belite clinker tends to show the same characteristics as that without SO_3_, and the strength of the 90-day net slurry can be above 100 Mpa, showing an upward trend. The cement clearly has good developmental benefits. In contrast, this kind of belite clinker requires more limestone and has a higher limestone saturation coefficient, but this is still about 10% lower than in ordinary Portland cement, which ensures its reaction with water and the formation of large amounts of silicate (Ca(OH)_2_). This increases the overall alkalinity, which speeds up the hydration process. In addition, the presence of 6% dihydrate gypsum had a positive effect on the development of the cement’s strength [48].

## 4. Conclusions

In this paper, a high belite cement clinker was synthesized from industrial raw materials and waste cement mortar. The influence of the calcination system and mineral composition design on clinker system synthesis was studied, the influence of waste mortar content on the composition of the clinker was explored, and the mechanism of the SO_3_ activation of belite was explored. The conclusions were as follows:

- The clinker was prepared by calcination at 1350 °C for one hour. It contained about 20~30% C_3_S, 50~70% C_2_S, 2~3% C_3_A, and 10~14% C_4_AF. Compared with ordinary silicate clinker, the contents of C_3_S and C_2_S were greatly reduced. This was modified by adding SO_3_ to the clinker to increase its early-phase strength. Different from belite-sulphoaluminate cement, it did not contain C_4_A_3_$;
- With the increase in calcination temperature or the extension of holding time, the content of C_3_S increased, and the content of C_2_S decreased. Hardened cement slurry can be used in waste mortar as the crystal seed, which is beneficial to the formation of alite. The addition of SO_3_ blocks the formation of C_3_S and stabilizes C_2_S;
- In the SO_3_ activation of belite, S^6+^ replaces Si^4+^, which increases the Ca/Si ratio in the belite, while also converting more Al into belite, such that the Al^3+^ replaces Si^4+^. Specifically, 5[SiO_4_]^4−^ replaces 3[AlO_4_]^5−^ and 2[SO_4_]^2−^. The increase in Ca/Si ratio and the solution of Al increases belite activity. At the same time, an increase in the Ca/Si ratio of belite leads to a decrease in alite content, and the presence of more Al in belite leads to a reduction in C_3_A content;
- The belite clinker was prepared by adding 6% dihydrate gypsum to make a cement paste with a low water–cement ratio (0.3). The cumulative 3-day heat release of the sample without SO_3_ was less than 120 J/g, and the 3-day strength was 13–18 MPa. The cumulative 3-day heat release of the sample with SO_3_ was less than 150 J/g, and the 3-day strength was 17–26 MPa. This material meets the requirements of low-heat cement, and the cement with SO_3_ showed better preliminary strength. The cement paste’s 90-day net strength was more than 100 MPa, indicating it has good later-phase strength.

These results indicate that waste concrete can be used to replace some raw materials, and can be added to SO_3_ to produce belite clinkers. Introducing this material into cement production can reduce the energy consumption of the cement industry, reduce the use of high-quality limestone, and reduce the carbon dioxide ranking. Furthermore, SO_3_ can also be replaced with waste raw materials with high SO_3_ contents.

## Figures and Tables

**Table 1:** Chemical composition of raw materials (wt.%).

Material	Loss	CaO	SiO_2_	MgO	Fe_2_O_3_	Al_2_O_3_	SO_3_
Limestone	40.6	51.65	0.816	0.753	0.08	0.05	0.027
Waste cement mortar	6.58	9.65	70.52	0.89	2.44	6.46	0.67
Coal gangue	18.4	0.98	51.02	0.7	3.83	21.65	0
Iron powder	1.16	4.68	58.6	3.63	26.93	3.2	0.21

**Table 2:** The composition of low waste mortar raw meals without the addition of SO_3_ in wt.% and expected basic chemical parameters.

Samples	Raw Material Mix/%				Chemical Composition/%				Modulus		
	Limestone	Coal Gangue	Waste Cement Mortar	Iron Powder	CaO	SiO_2_	Al_2_O_3_	Fe_2_O_3_	KH	SM	IM
A1	72.80	8.50	9.10	9.60	57.16	24.68	4.16	4.75	0.70	2.78	0.88
A2	73.30	8.30	9.00	9.40	57.64	24.31	4.09	4.66	0.72	2.79	0.88
A3	73.70	8.20	8.80	9.30	58.02	23.99	4.04	4.62	0.74	2.78	0.87
A4	74.10	8.20	8.60	9.10	58.41	23.67	4.02	4.55	0.75	2.77	0.88
A5	74.50	8.20	8.20	9.10	58.78	23.30	3.99	4.54	0.77	2.74	0.88

**Table 3:** The composition of high waste mortar raw meals without the addition of SO_3_ in wt.% and expected basic chemical parameters.

Samples	Raw Material Mix/%				Chemical Composition/%				Modulus		
	Limestone	Coal Gangue	Waste Cement Mortar	Iron Reagent	CaO	SiO_2_	Al_2_O_3_	Fe_2_O_3_	KH	SM	IM
B1	72.70	7.40	17.40	2.50	57.75	24.25	4.16	4.83	0.72	2.70	0.86
B2	73.10	7.40	17.10	2.40	58.13	24.00	4.14	4.69	0.74	2.72	0.88
B3	73.70	7.20	16.70	2.40	58.69	23.50	4.05	4.68	0.76	2.70	0.87
B4	74.10	7.00	16.50	2.40	59.06	23.18	3.97	4.67	0.78	2.69	0.85

**Table 4:** The composition of raw meals with the addition of 2 and 6 wt.% GY in wt.% and expected basic chemical parameters.

Samples	Raw Material Mix/%					Chemical Composition/%				Modulus		
	Limestone	Coal Gangue	Waste Cement Mortar	Iron Reagent	CaSO_4_∙2H_2_O	CaO	SiO_2_	Al_2_O_3_	Fe_2_O_3_	KH	SM	IM
C1	72.10	7.20	16.40	2.30	2.00	57.75	24.25	4.16	4.83	0.76	2.72	0.89
C2	72.90	6.90	15.90	2.30	2.00	58.13	24.00	4.14	4.69	0.79	2.69	0.86
C3	69.60	6.60	15.60	2.20	6.00	58.69	23.50	4.05	4.68	0.76	2.73	0.89
C4	72.40	6.20	15.20	2.20	6.00	59.06	23.18	3.97	4.67	0.80	2.71	0.85

**Table 5:** ICSD numbers of mineral phase.

Phase	ICSD Number
C_4_AF	9197 [29]
C_3_A	1841 [30]
C_2_S	79,551 [31]
C_3_S	22,501 [32]
CaO	75,785 [33]

**Table 6:** Phase composition (wt.%) of A1–A4 sintered at 1350 °C, for 30 min.

		C_2_S	C_3_S	C_4_AF
A1	Rietveld	70.1	8.6	11.8
	XRF	74.4	9.5	13.3
A2	Rietveld	63.5	13.4	11.7
	XRF	68.9	14.6	13.6
A3	Rietveld	58.1	17.6	11.7
	XRF	65.4	19.6	13.2
A4	Rietveld	53.4	23.9	11.2
	XRF	59.4	23.8	13.9
A5	Rietveld	39.3	27.3	12.3
	XRF	54.8	28.8	13.5

**Table 7:** Phase composition (wt.%) of B1–B4 sintered at 1350 °C, for 30 min.

		C_2_S	C_3_S	C_4_AF
B1	Rietveld	68.3	15.4	11.6
	XRF	68.5	14.8	13.8
B2	Rietveld	59.1	20.2	11.1
	XRF	64.7	18.7	13.7
B3	Rietveld	52.0	24.8	9.6
	XRF	58.9	24.7	13.7
B4	Rietveld	43.4	29.9	8.9
	XRF	53.5	29.8	13.9

**Table 8:** Phase composition (wt.%) of C1–C4 sintered at 1350 °C, for 30 min.

		C_2_S	C_3_S	C_4_AF
C1	Rietveld	46.6	21.6	12.3
	XRF	56.1	24.4	12.8
C2	Rietveld	41.3	26.3	12.3
	XRF	50.4	28.8	13.7
C3	Rietveld	54.2	17.0	10.2
	XRF	54.9	23.6	12.9
C4	Rietveld	44.2	23.1	12.7
	XRF	48.4	29.2	13.1

**Table 9:** Average chemical composition of belite grains in clinkers (wt. %).

Clinker	B1	B2	B3	B4	C1	C2	C3	C4
CaO	62.86	62.56	62.84	62.12	63.56	63.31	62.15	63.16
SiO_2_	31.12	31.21	30.95	30.42	27.56	28.38	25.96	25.94
Al_2_O_3_	1.56	1.21	1.51	1.83	2.5	2.45	2.76	2.69
Fe_2_O_3_	2.18	2.41	2.25	2.17	2.13	2.15	2.13	2.11
SO_3_	0.31	0.45	0.3	0.3	1.25	1.54	2.35	2.61
MgO	0.37	0.21	0.31	0.21	0.23	0.34	0.3	0.3
K_2_O	0.4	0.6	0.61	0.9	0.63	0.92	0.91	0.91
Na_2_O	0.62	0.42	0.61	0.6	0.63	0.31	0.61	0.3
TiO_2_	0.03	0	0	0.06	0.07	0	0.05	0.04
P_2_O_5_	0.34	0.3	0.4	0.9	0.63	0.31	0.91	0.6
Ca/Si(atomic)	2.164	2.148	2.175	2.188	2.471	2.390	2.565	2.609
(Ca + k + Na + Mg): (Si + S + P + Al + Fe + Ti) (atomic)	1.982	1.967	1.994	1.964	2.096	2.045	2.086	2.105

**Table 10:** The correlation matrix of the average atomic ratio values of sets of belites from eight experimental samples with different input sulfur content and lime saturation values. The critical value of Pearson’s correlation coefficient is ±0.703 (ν = 8, α = 0.05, two-tailed); significant values are typed bold (significant positive correlation) or bold italics (significant negative correlation).

	Na	K	Mg	Ca	Si	Al	S	P	Fe
Na	1								
K	−0.45	1							
Mg	−0.12	−0.15	1						
Ca	−0.341	0.193	0.259	1					
Si	0.281	−0.644	-0.094	−0.218	1				
Al	−0.249	**0.719**	0.203	0.227	***−0.946***	1			
S	−0.458	0.651	0.196	0.196	***−0.967***	**0.893**	1		
P	0.386	0.564	-0.379	−0.512	−0.498	0.525	0.356	1	
Fe	−0.051	−0.456	-0.369	−0.26	0.698	***−0.85***	−0.594	−0.515	1

## Data Availability

Not applicable.
